# The perception of behaviour associated with dementia in the acute hospital

**DOI:** 10.1192/bjo.2021.693

**Published:** 2021-06-18

**Authors:** Zumer Jawaid, George Crowther, Syeda Ashar

**Affiliations:** 1 Leeds and York Partnership Foundation Trust; 2 Leeds Teaching Hospitals Trust

## Abstract

**Aims:**

General hospital based Health Care Professionals (HCPs) use very varied language to describe behaviour in dementia. Lessons from medicine and other professions tell us that non-uniform communication is a source of error and subsequent poor decision making. Knowing how HCPs communicate behaviour in dementia in a hospital setting may help better understand these potential sources of communication error and identify training needs.

**Background:**

Around 25% of hospital beds occupied with people living with dementia. Hospitalised patients with dementia have a high prevalence of distressing symptoms (pain 70%, delirium 66%, depression 35%, anxiety 34%, hallucinations 14% delusions 11%). These symptoms often displayed as behaviour can be challenging for HCPs to interpret. Variations in communicating behaviour may lead to inconsistent understanding of the need, with the potential for missing treatable conditions that drive the behaviour. Standardizing communication and documentation have the potential to improve the quality of information handed over between HCPs which may improve the quality of care and patient outcomes.

**Method:**

Qualitative methodology including photo elicitation was used. A purposive sample of 59 HCPs was selected. This was identified from a range of professional backgrounds, experience levels and medical specialities. They were presented with a photograph and case vignettes depicting 4 behaviours associated with distress (aggression, depression, delirium and psychosis). HCPs were asked to respond to the scenarios as if they were handing over to colleagues or documenting in the medical record. Data were analysed by thematic analysis.

**Result:**

59 HCPs were interviewed with photo-elicitation. Participants recorded their responses in limited time to reflect time constraints in a busy ward environment. 2 HCPs declined to participate in research.

When describing behaviour associated with aggression and depression HCPs were consistent with the language used (49/57). When presented with a delirium less consistency was observed (31/47). While describing psychosis each HCP chose either paranoia or suspiciousness among other descriptions.

**Conclusion:**

Overall there has been consistency in describing the distress experienced by the patient even though HCPs came from very different roles and specialities. Doctors, Nurses, CSWs and dieticians all described the behaviour alike. Newer staff were more accurate which could be due to dementia training within National Dementia Action Alliance.

